# NT-ProBNP and mortality across the spectrum of glucose tolerance in the general US population

**DOI:** 10.1186/s12933-022-01671-w

**Published:** 2022-11-07

**Authors:** Stefano Ciardullo, Federico Rea, Rosa Cannistraci, Emanuele Muraca, Silvia Perra, Francesca Zerbini, Andrea Mortara, Gianluca Perseghin

**Affiliations:** 1 Department of Medicine and Rehabilitation, Policlinico di Monza, Via Modigliani 10, 20900 Monza, Italy; 2 School of Medicine and Surgery, Department of Medicine and Surgery, University of Milano Bicocca, Milan, Italy; 3 National Centre for Healthcare Research and Pharmacoepidemiology, at the University of Milano-Bicocca, Milan, Italy; 4 Laboratory of Healthcare Research and Pharmacoepidemiology, Unit of Biostatistics, Epidemiology and Public Health, Department of Statistics and Quantitative Methods, University of Milano-Bicocca, Milan, Italy; 5 Clinical Cardiology, Policlinico di Monza, Monza, Italy

**Keywords:** NT-ProBNP, Mortality, Diabetes, Epidemiology

## Abstract

**Background:**

Even though hyperglycemia is a well-known cardiovascular risk factor, the absolute risk of cardiovascular events varies to a great extent within each glycemic category. The aim of this study is to evaluate whether N-terminal pro-B natriuretic peptide (NT-ProBNP) could help identify subjects at higher cardiovascular risk, independently of blood glucose levels.

**Methods:**

Serum NT-ProBNP levels were measured in 5502 people aged 45–79 years without heart failure from the general population (3380 with normoglycemia, 1125 with pre-diabetes and 997 with diabetes) that participated in the 1999–2004 cycles of the National Health and Nutrition Examination Survey. We applied Cox and Fine Gray models adjusted for cardiovascular risk factors to evaluate the association between NT-ProBNP levels and all-cause and cardiovascular mortality through December 2015.

**Results:**

After a median follow-up of 13 years, 1509 participants died, 330 of cardiovascular causes. In the multivariable-adjusted models, compared with participants with NT-ProBNP < 100 pg/ml, those with levels 100–300 pg/ml and ≥ 300 pg/ml had a higher incidence of both all-cause mortality (HR 1.61, 95% CI 1.12–2.32, p = 0.012 and HR 2.96, 95% CI 1.75–5.00, p < 0.001, respectively) and cardiovascular mortality (HR 1.57, 95% CI 1.17–2.10, p = 0.011 and HR 2.08, 95% CI 1.47–2.93, p < 0.001, respectively). The association was consistent in subgroup analyses based on glycemic status, obesity, age and sex.

**Conclusions:**

Elevated NT-ProBNP is independently associated with all-cause and cardiovascular mortality in the general population and could help identify patients at the highest risk. Further studies are needed to evaluate whether intensification of treatment based on biomarker data might lead to improvements in cardiovascular risk reduction.

**Supplementary Information:**

The online version contains supplementary material available at 10.1186/s12933-022-01671-w.

## Background

Large observational studies have shown a strong association between blood glucose levels and incident coronary artery disease, stroke, heart failure, as well as all-cause and cardiovascular mortality [1].

Nonetheless, especially for patients without prevalent cardiovascular disease, the risk of adverse clinical outcomes is highly heterogeneous [2]. Risk stratification is of paramount importance in this patient population, as the predicted risk might inform treatment strategies [3]. For patients with diabetes, according to recent guidelines from the American Diabetes Association (ADA), the European Association for the Study of Diabetes (EASD) and the European Society of Cardiology (ESC), presence of cardiovascular disease (or high risk for cardiovascular events) has a crucial role in informing treatment [4–6]. In particular, patients at high risk should be treated with cardio-protective agents such as glucagon- like peptide 1 receptor agonists (GLP1-RA) or sodium-glucose transporter 2 inhibitors (SGLT2-i). In patients without prevalent cardiovascular disease, ADA guidelines suggest using the American College of Cardiology/American Heart Association ASCVD risk calculator (Risk Estimator Plus) to risk-stratify patients [7]. This process does not only inform future glucose lowering treatment, but also anti-hypertensive and lipid lowering therapy, suggesting more stringent targets in patients at higher risk. Nonetheless, performance of risk stratification tools remains sub-optimal [8, 9], which justifies the search of new biomarkers that may add to the prediction offered by available methods. However, previous studies showed contrasting results [10–12]. Recently, a biomarker that has received attention is N-terminal pro-B natriuretic peptide (NT-ProBNP), a cardiac hormone, secreted by cardiomyocytes in response to an increase in ventricular wall stretch [13]. It has been shown to predict the risk and the severity of heart failure, and for these reasons is now used almost routinely for the diagnosis and management of subjects with or at high risk for heart failure [14, 15]. The predictive value of NT-ProBNP has also been shown in few community studies [16–19], as well as in some clinical conditions such as hypertension and coronary artery disease (CAD) [20]. Fewer studies evaluated its association with outcomes in analyses stratified by glycemic status [21]. It has been previously shown that increasing age, kidney dysfunction and elevated BMI might impact NT-proBNP and reduce its ability to accurately predict heart failure [22, 23]. Since the prevalence of all these aspects varies substantially going from normal glucose tolerance to pre-diabetes and diabetes, the aim of the present study is to evaluate whether the association between NT-proBNP levels and hard clinical outcomes varies according to the glycemic status.

To achieve this goal, we analyzed data obtained from the 1999–2004 cycles of the National Health and Nutrition Examination Survey (NHANES) and evaluated the association between NT-ProBNP, glucose levels and all-cause and cardiovascular mortality in people without heart failure from the general population of the United States.

## Methods

All data used in the current analysis are publicly available through the National Center for Health Statistics and can be accessed at https://wwwn.cdc.gov/nchs/nhanes/default.aspx.

This is an analysis of data from the 1999–2004 cycles of NHANES, which is conducted in the United States by the National Center for Health Statistics. It is an ongoing cross-sectional complex survey aimed at including individuals representative of the general, non-institutionalized population of all ages. To this end, it applies a stratified, multistage, clustered probability sampling design with oversampling of non-Hispanic black and Hispanic persons, people with low income and older adults. The survey consists of a structured interview conducted in the home, followed by a standardized health examination that includes a physical examination as well as laboratory tests. Full methodology of data collection is available elsewhere [24]. The original survey was approved by the Centers for Disease Control and Prevention Research Ethics Review Board and written informed consent was obtained from all adult participants. The present analysis was deemed exempt by the Institutional Review Board at our institution, as the dataset used in the analysis was completely de-identified.

### Glycemic measures

Hemoglobin HbA1c was measured in whole blood as part of the original NHANES 1999–2004 protocols at the University of Missouri-Columbia using high performance chromatography on the Primus CLC 330 and Primus CLC 385 instruments (Primus Corporation, Kansas City, Missouri, USA). The laboratory procedure manual is available at the NHANES website [25]. Participants were considered having diabetes if they answered positively to the following question: “Other than during pregnancy, have you ever been told by a doctor or health professional that you have diabetes or sugar diabetes?” and/or if they met at least two of the following criteria: HbA1c ≥ 6.5%, random plasma glucose ≥ 200 mg/dl, fasting plasma glucose ≥ 126 mg/dl [26]. As not all participants were evaluated after an eight hour fast, we divided participants without diabetes into the normoglycemia and pre-diabetes groups based on HbA1c levels < 5.7% and between 5.7 and 6.4%, respectively [27].

### Laboratory tests and clinical data

Participants self-reported age, sex, race-ethnicity (categorized as non-Hispanic white, non-Hispanic black, Hispanic or other), education, smoking status and previous medical history. Body measurements including height (cm), weight (kg) and waist circumference (cm) were ascertained during the mobile examination center visit; BMI was calculated as weight in kilograms divided by height in meters squared.

Alcohol consumption was estimated based on self-reported data on the amount and frequency of alcohol use within the previous year. It was considered significant if > 1 drink per day for women and > 2 drinks per day for men on average [28].

Laboratory methods for measurements of glucose, lipid profile, ALT, AST, creatinine, cholesterol and triglycerides levels are reported in detail elsewhere [29].

Estimated glomerular filtration rate (eGFR) was computed according to the Chronic Kidney Disease Epidemiology Collaboration (CKD-EPI) equation and CKD was defined as an eGFR < 60 ml/min/1.73 m^2^. Based on the measured urine albumin to creatinine ratio (UACR) participants were defined as having normo-albuminuria (UACR < 30 mg/g), micro-albuminuria (UACR between 30 and 300 mg/g) or macro-albuminuria (UACR ≥ 300 mg/dl) [30]. Diagnoses of heart failure (HF), coronary artery disease (CAD) and stroke were based on self-report. All patients reporting a previous history of heart failure were excluded from the present study. Hypertension was defined according to the 2018 European Society of Cardiology (ESC)/European Society of Hypertension (ESH) Guidelines as a SBP value ≥ 140 mmHg and/or a DBP value ≥ 90 mmHg or currently taking antihypertensive drugs [31]. The remaining participants were segregated in the following 3 groups: optimal BP (SBP < 120 mmHg and DBP < 80 mmHg), normal BP (SBP 120–129 mmHg and/or DBP 80–84 mmHg) and high normal BP (SBP 130–149 mmHg and/or DBP 85–89 mmHg) [32, 33].

### NT-proBNP measurement

Sera from stored surplus specimens from NHANES 1999–2004 participants were tested during 2018–2020 at the University of Maryland School of Medicine, Baltimore, Maryland. NT-proBNP was measured in serum on the Roche e611 autoanalyzer. The lower and upper limits of detection are 5 pg/ml and 35000 pg/ml, respectively.

The CV of NT-ProBNP was 3.1% (low, 46 pg/ml) and 2.7% (high, 32805 pg/ml).

Only sera samples that had not previously undergone freeze–thaw (i.e., pristine samples) were used in the present study. In this study, NT-ProBNP was analyzed as a continuous variable as well as categorized according to pre-specified cut-offs. Based on previous analyses, NT-ProBNP was categorized into pre-specified levels of < 100, 100 to 299 and ≥ 300 pg/ml [14].

### All-cause and cardiovascular mortality

Mortality data from death certificates from the National Death Index were linked to NHANES based on the participant sequence number available on both datasets. The present analysis is based on the public-use linked mortality files for NHANES 1999–2004, which include follow-up time and cause of death for adult participants through 31 December 2015. Cardiovascular mortality was defined as death due to heart disease or cerebrovascular disease, as reported on death certificate records. More information on the linkage method and analytic guidelines are available on the specific webpage of the National Center for Health Statistics [34].

### Analysis sample

Among a total of 7173 participants 45–79 years, 6709 attended a mobile examination center visit. We initially excluded 830 individuals without an available NT-ProBNP measurement, leading to a population of 5879. Among these, 377 were excluded because of prevalent heart failure or missing data on HbA1c, leading to a final sample of 5502 participants (Additional file 1: Fig. S1).

### Statistical analysis

All analyses were conducted using Stata version 16 (StataCorp, College Station, TX), accounting for the complex survey design of NHANES. We used appropriate weighting for each analysis, as suggested by the NCHS to obtain estimates that were generalizable to the U.S. population aged 45–79 years. Data are expressed as weighted proportions (Standard Error (SE)) for categorical variables and as weighted means (SE) for continuous variables. Participants’ characteristics according to NT-ProBNP levels were compared using linear regression for continuous variables and the design-adjusted Rao-Scott chi-square test for categorical variables.

The overall incidence of death at a given time was decomposed into a sum of the individual cumulative incidence functions for cardiovascular and non-cardiovascular mortality [35].

Cox proportional hazard model and Fine and Gray model were applied to evaluate the association between NT-ProBNP and all-cause and cardiovascular mortality, respectively. We verified the proportional hazard assumption by using log–log plots and by testing for interaction by log(time). The following potential confounders, selected based on clinical evaluation, were included in the models: age, sex, race-ethnicity, BMI, education, cigarette smoke, total cholesterol, eGFR, diabetes and alcohol consumption.

Analyses were separately performed for participants with normoglycemia, pre-diabetes, and diabetes. In addition, analyses were repeated after stratification for sex, obesity status, and age. An interaction term between NT-proBNP and strata was included in the model to test whether the NT-proBNP has a different effect between groups.

To evaluate the robustness of our findings, analyses were repeated after the exclusion of patients with chronic kidney disease.

A two-tailed value of p < 0.05 was considered statistically significant.

## Results

### Features of the study population

The study population consisted in 3380 participants with normoglycemia, 1125 with pre-diabetes and 997 patients with diabetes (weighted prevalence 13.4%). Clinical and biochemical features of the whole population divided by NT-ProBNP levels are shown in Table 1. Participants with higher NT-ProBNP were older, more frequently female, had higher SBP and DBP levels and less frequently current smokers. They also had lower eGFR and higher HbA1c levels. Most comorbid conditions were more prevalent in participants with higher NT-ProBNP levels, including hypertension, diabetes and albuminuria, while no significant difference was found in BMI.

**Table 1:** Baseline characteristics of participants according to NT-ProBNP levels

NT-ProBNP (pg/ml)				
Variable	< 100	100–299	≥ 300	Total
Age (years)	55.1 (0.2)	61.8 (0.4)	66.3 (0.6)	57.4 (0.2)
Age category (%)				
45–59	73.2 (1.0)	42.1 (1.8)	24.5 (2.4)	62.5 (1.1)
60–79	26.8 (1.0)	57.9 (1.8)	75.5 (2.4)	37.5 (1.1)
Male (%)	53.0 (1.0)	26.5 (1.2)	36.3 (2.8)	45.5 (0.7)
Race-ethnicity (%)				
Non-Hispanic White	75.3 (1.9)	81.5 (2.0)	81.4 (2.8)	77.2 (1.8)
Hispanic	10.1 (1.7)	8.1 (1.5)	7.8 (2.0)	9.5 (1.6)
Non-Hispanic Black	9.9 (1.0)	7.3 (1.0)	9.6 (1.7)	9.3 (0.9)
Other	4.7 (0.7)	3.0 (0.7)	1.2 (0.6)	4.0 (0.6)
BMI (kg/m^2^)	28.9 (0.1)	28.1 (0.2)	28.5 (0.5)	28.6 (0.1)
SBP (mmHg)	127.2 (0.3)	135.0 (0.8)	143.6 (1.5)	130.2 (0.3)
DBP (mmHg)	75.6 (0.2)	72.6 (0.4)	72.9 (0.8)	74.7 (0.2)
Triglycerides (mg/dL)	156.5 (3.1)	132.4 (2.6)	146.1 (5.3)	150.0 (2.3)
HbA1c (%)	5.7 (0.0)	5.6 (0.0)	5.7 (0.1)	5.6 (0.0)
HDL-C (mg/dL)	52.3 (0.3)	58.0 (0.6)	56.5 (1.2)	53.9 (0.3)
AST (U/L)	25.8 (0.3)	24.0 (0.5)	25.5 (0.7)	25.3 (0.2)
ALT (U/L)	27.4 (0.4)	21.9 (0.6)	22.5 (1.1)	25.8 (0.3)
eGFR (ml/min/1.73 m^2^)	91.5 (0.3)	85.0 (0.6)	73.2 (1.4)	88.8 (0.3)
Total Cholesterol (mg/dl)	212.1 (0.8)	209.2 (1.3)	206.6 (2.7)	211.0 (0.7)
Blood pressure (%)				
Optimal	24.9 (1.1)	19.5 (1.3)	7.8 (1.9)	22.5 (0.9)
Normal	20.1 (0.9)	12.7 (1.1)	8.9 (1.9)	17.6 (0.7)
High-normal	13.6 (0.6)	12.2 (1.2)	10.9 (1.9)	13.1 (0.5)
Hypertension	41.4 (1.4)	55.6 (1.6)	72.3 (2.3)	46.9 (1.2)
Education (%)				
High school or less	43.0 (1.4)	49.2 (1.9)	62.8 (3.9)	45.8 (1.3)
Some college	29.8 (1.0)	27.1 (1.7)	19.9 (2.4)	28.5 (0.9)
College graduate or above	27.2 (1.4)	23.7 (1.8)	17.3 (3.1)	25.7 (1.2)
Glycemic category (%)				
Euglycemic	70.0 (1.4)	69.9 (2.1)	58.6 (2.0)	69.0 (1.2)
Pre-diabetes	17.7 (1.0)	16.9 (1.7)	18.9 (1.7)	17.6 (0.8)
Diabetes	12.3 (0.7)	13.1 (1.1)	22.5 (1.9)	13.4 (0.6)
UACR (mg/g, %)				
< 30	92.0 (0.6)	87.8 (1.3)	66.1 (2.6)	89.4 (0.6)
30–300	7.3 (0.5)	10.8 (1.2)	25.3 (2.4)	9.3 (0.5)
> 300	0.7 (0.2)	1.4 (0.3)	8.6 (1.3)	1.4 (0.2)
Cigarette smoke (%)				
Never	44.9 (1.2)	49.6 (1.7)	42.0 (2.6)	45.8 (1.0)
Former	34.4 (1.0)	31.7 (1.7)	40.2 (2.6)	34.1 (1.0)
Current	20.7 (0.9)	18.8 (1.4)	17.7 (2.1)	20.1 (0.8)

Data are expressed as weighted proportions (Standard Error (SE)) for categorical variables and as weighted means (SE) for continuous variables

*BMI* Body Mass Index, *CKD* chronic kidney disease, *CVD* cardiovascular disease, *UACR* urinary albumin creatinine ratio, *HbA1c* Hemoglobin A1c, *HDL* high density lipoprotein, *SBP* systolic blood pressure, *DBP* diastolic blood pressure

### NT-ProBNP and mortality

Over a median follow-up of 13 years, 1509 participants (415 with and 1094 without diabetes) died, 330 of cardiovascular causes. Cumulative incidence functions of cardiovascular and non-cardiovascular death in the whole population and according to glucose tolerance are reported in Additional file 1: Fig. S2, while incidence rates of all-cause mortality (for 1000 person-years) and cardiovascular mortality in each NT-ProBNP category are shown in Table 2. They increased proportionally with increasing NT-ProbBNP levels in the overall population, as well in each blood glucose group.

**Table 2:** Number of events and incidence rates for all-cause and cardiovascular mortality in US adults by NT-ProBNP values

NT-ProBNP (pg/ml)				
	All-cause mortality		Cardiovascular mortality	
	Events (n/N)	Incidence rate per 1000 person-years (95% CI)	Events (n/N)	Incidence rate per 1000 person-years (95% CI)
Entire cohort				
< 100	655/3446	14.8 (13.7–15.9)	123/3446	2.8 (2.3–3.3)
100–299	499/1462	29.0 (26.6–31.7)	116/1462	6.7 (5.6–8.1)
≥ 300	355/585	64.2 (57.8–71.2)	91/585	16.5 (13.4–20.2)
HbA1c < 5.7%				
< 100	323/2144	11.5 (10.3–12.9)	59/2144	2.1 (1.6–2.7)
100–299	284/925	25.3 (22.6–28.5)	53/925	4.7 (3.6–6.2)
≥ 300	169/305	55.6 (47.8–64.6)	33/305	10.8 (7.7–15.3)
HbA1c 5.7–6.4%				
< 100	151/727	16.5 (14.0–19.3)	24/727	2.6 (1.8–3.9)
100–299	92/280	28.4 (23.1–34.7)	27/280	8.3 (5.7–12.2)
≥ 300	75/116	69.9 (55.7–87.6)	22/116	20.5 (13.5–31.1)
Diabetes				
< 100	181/575	25.7 (22.2–29.8)	40/575	5.7 (4.2–7.8)
100–299	123/257	44.7 (37.5–53.3)	36/257	13.1 (9.4–18.1)
≥ 300	111/164	78.4 (65.1–94.4)	36/164	25.4 (18.3–35.2)

*NT-ProBNP* N-terminal pro-B natriuretic peptide

**Fig. 1:**
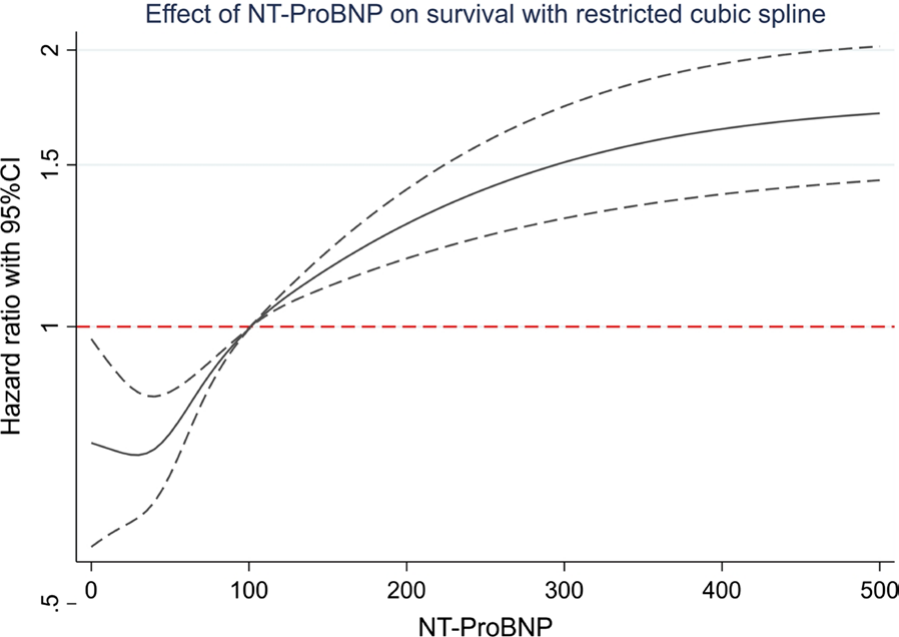
Adjusted hazard ratios (HRs; 95% confidence intervals) for baseline NT-proBNP with all-cause mortality. Adjusted HRs are from Cox proportional hazards models. Baseline NT-proBNP levels were modeled using restricted cubic splines (solid lines)

The effect of NT-ProBNP on survival estimated by restricted cubic spline is shown in Fig. 1. The mortality risk increased as NT-ProBNP increased.

The hazard ratios evaluating the association between NT-ProBNP levels and all-cause mortality in the whole population and according to glycemic status are shown in Table 3. In all groups, higher NT-ProBNP levels were significantly associated with increased risks of all-cause mortality.

**Table 3:** Cox-proportional hazard model evaluating the association between NT-ProBNP levels and all-cause mortality according to baseline HbA1c levels

Population	NT-ProBNP (pg/ml)	All-cause mortality		
		HR	95% CI	p-value
Whole population	Per 1 SD	1.11	1.07–1.17	< 0.001
	< 100	1.00	Ref.	
	100–299	1.61	1.12–2.32	0.012
	≥ 300	2.96	1.75–5.00	< 0.001
HbA1c < 5.7%	Per 1 SD	1.12	1.07–1.18	< 0.001
	< 100	1.00	Ref.	
	100–299	1.72	1.00–2.95	0.050
	≥ 300	3.12	1.50–6.49	0.003
HbA1c 5.7–6.4%	Per 1 SD	1.23	1.05–1.43	0.010
	< 100	1.00	Ref.	
	100–299	1.84	0.92–3.67	0.082
	≥ 300	3.79	1.59–9.01	0.003
Diabetes	Per 1 SD	1.10	1.05–1.16	< 0.001
	< 100	1.00	Ref.	
	100–299	1.44	0.81–2.56	0.206
	≥ 300	2.43	0.98–6.06	0.056

Covariates included in model were age, sex, race-ethnicity, body mass index, education, cigarette smoke, total cholesterol, eGFR, blood pressure category, prevalent cardiovascular disease and alcohol consumption

*CI* confidence interval, *NT-ProBNP* N-terminal pro-B natriuretic peptide

As shown in Table 4, higher NT-ProBNP levels were also significantly associated with an increased risk of cardiovascular death when the whole population was considered. Positive trends were identified in all groups, while statistical significance was not achieved in patients with diabetes.

**Table 4:** Fine and Gray model evaluating the association between NT-ProBNP levels and cardiovascular mortality according to baseline HbA1c levels

Population	NT-ProBNP (pg/ml)	Cardiovascular mortality		
		HR	95% CI	p-value
Whole population	Per 1 SD	1.07	1.02–1.13	0.009
	< 100	1.00	Ref	
	100–299	1.57	1.17–2.10	0.003
	≥ 300	2.08	1.47–2.93	< 0.001
HbA1c < 5.7%	Per 1 SD	1.17	1.07–1.28	0.001
	< 100	1.00	Ref	
	100–299	1.35	0.87–2.10	0.186
	≥ 300	1.79	1.05–3.05	0.032
HbA1c 5.7–6.4%	Per 1 SD	1.07	0.85–1.35	0.577
	< 100	1.00	Ref	
	100–299	2.15	1.20–3.85	0.010
	≥ 300	3.04	1.47–6.28	0.003
Diabetes	Per 1 SD	1.03	0.96–1.10	0.406
	< 100	1.00	Ref	
	100–299	1.64	0.98–2.75	0.061
	≥ 300	1.81	0.99–3.34	0.055

Covariates included in model were age, sex, race-ethnicity, body mass index, education, cigarette smoke, total cholesterol, eGFR, blood pressure category, prevalent cardiovascular disease and alcohol consumption

*CI* confidence interval, *NT-ProBNP* N-terminal pro-B natriuretic peptide

**Fig. 2:**
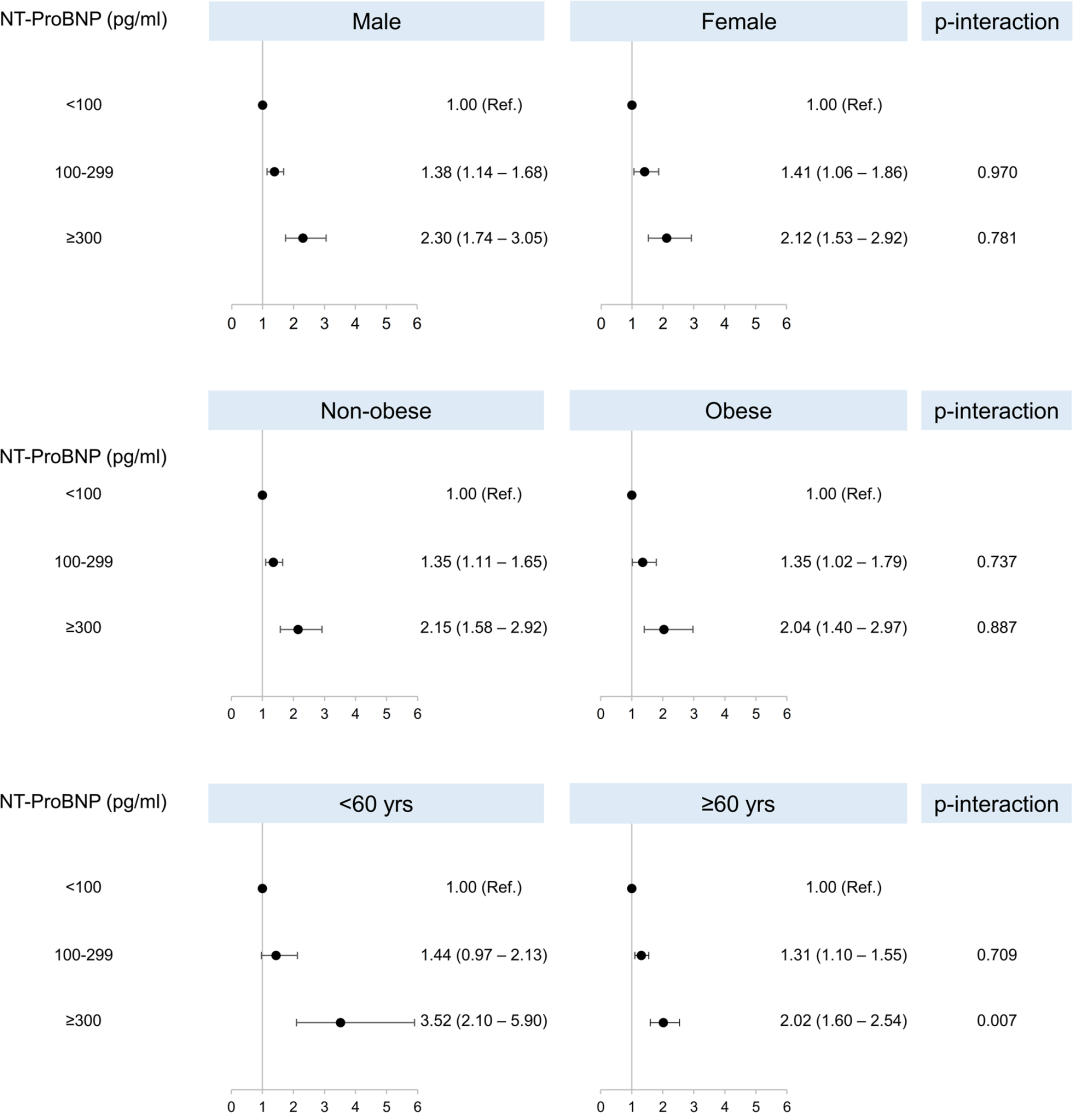
Hazard ratios and 95% confidence intervals for all-cause mortality according to NT-ProBNP leves in subgroups of the overall population. Estimates were obtained with a Cox proportional hazard model adjusted for age, sex, race-ethnicity, body mass index, education, cigarette smoke, total cholesterol, chronic kidney disease, diabetes and alcohol consumption

We then performed subgroup-sensitivity analysis to evaluate the consistency of our findings. As shown in Fig. 2, higher levels were associated with increased incidence of all-cause mortality after adjustment for potential confounders independently of age, sex and presence of obesity. However, there was evidence that NT-ProBNP ≥ 300 pg/ml had a higher impact on survival among younger rather than older patients (p = 0.007).

The main results were confirmed after the exclusion of patients with chronic kidney disease (Additional file 1: Table S1).

## Discussion

In the present population-based cohort study, performed on a large and representative sample of the United States population, we made a series of observations. First, we found that NT-ProBNP levels were significantly associated with all-cause and cardiovascular mortality after a median follow-up of 13 years in people without a previous history of heart failure. Second, this association was consistent after adjustment for known cardiovascular risk factors and socio-behavioral factors. Third, NT-ProBNP was associated with an increased risk of both all-cause and cardiovascular mortality to a similar extent in people with normo-glycemia, pre-diabetes and diabetes. Fourth, results were consistent in analyses stratified by age, sex and presence of obesity, with higher HRs being present for older individuals.

The prognostic value of NT-ProBNP for cardiovascular outcomes in patients with diabetes has been demonstrated in several studies [36]. Recently, Prausmüller et al. compared the performance of NT-ProBNP with the ESC/EASD risk model and the Systemic Coronary Risk Evaluation in predicting cardiovascular and all-cause mortality in 1690 unselected patients with type 2 diabetes [37]. The authors showed that NT-ProBNP alone was superior to these models in predicting all outcomes, with consistent results across age groups, making a case for a more widespread use of biomarkers to risk-stratify patients with diabetes.

On the other hand, few studies investigated whether the association between NT-ProBNP and outcomes differed according to glycemic status. Nguyen et al. studied 5584 participants aged 45 to 84 years from the Multi-Ethnic Study of Atherosclerosis of which 4090 were normoglycemic, 799 had prediabetes, and 695 had diabetes at baseline. The Authors showed that rates of heart failure and cardiovascular disease increased progressively at higher NT-ProBNP levels. Moreover, addition of this biomarker to conventional risk factors significantly improved risk prediction. The predictive ability of the biomarker did not differ significantly according to glucose status [21]. The present study corroborates these results, while also demonstrating consistency in analyses stratified by age, sex and body mass index.

Interestingly, the PONTIAC (NT-ProBNP Guided Primary Prevention of CV Events in diabetic patients) trial showed that measuring NT-ProBNP levels could be a way to identify patients with diabetes in primary cardiovascular prevention that might benefit more from treatment with renin–angiotensin–aldosterone blockers and beta-blockers [20]. In this context, our study contributes to the growing evidence supporting the use of biomarkers to inform therapeutic choices. Ultimately, prospective controlled clinical trials would be required to evaluate whether such an approach to guide treatment would result in net clinical benefits for cardiovascular prevention.

Our study has several strengths. It is a large study performed in an unselected sample of US adults with all glycemic status groups being represented, including both sexes and patients of different ethnic background. The high number of participants included yielded high statistical power to perform sensitivity analyses and evaluate the impact of several variables in multivariable models. Being based on NHANES data, our results have a high degree of external validity as the purpose of the survey is to be representative of the overall US population. Acquisition of clinical, laboratory and anthropometric data was standardized and homogenous. Finally, we focused on hard clinical outcomes with very high degree of retention of participants, as data were based on the National Death Index.

On the same lines, several limitations should also be acknowledged. First, we can only provide evidence on mortality, while data on non-fatal cardiovascular events are not available. Second, all variables were measured at a single baseline examination and no data are available on longitudinal changes in NT-ProBNP levels, leading to the possibility of misclassification. Third, data on certain variables, including smoking, alcohol use and prevalent cardiovascular disease and heart failure were based exclusively on self-report. It is possible that some patients with heart failure were not aware of their condition and were therefore included in the study. The lack of echocardiographic data did not allow us to verify this hypothesis. Finally, despite adjustment for several risk factors for mortality, the possibility of residual confounding cannot be completely excluded.

## Conclusions

In conclusion, we show that higher NT-ProBNP levels are independently associated with all-cause mortality not only in people without heart failure, irrespectively of blood glucose levels, sex, age and body mass index. These findings add to the growing evidence for the utility of measuring NT-ProBNP to risk-stratify patients and guide treatment. Further large-scale studies testing this hypothesis are required.

## Supplementary Information

**Additional file 1: Fig. S1.** Flow-chart of the studied participants. **Fig. S2.** Cumulative incidence of cardiovascular and non-cardiovascular mortality in the whole population and according to glucose tolerance. **Table S1.** Cox-proportional hazard model evaluating the association between NT-ProBNP levels and all-cause mortality according to baseline HbA1c levels by excluding patients with chronic kidney disease.

## Data Availability

All data analyzed during this study are publicly available at the NHANES website.
