# Supplementary material for: NT-ProBNP and mortality across the spectrum of glucose tolerance in the general US population
Source: Cardiovasc Diabetol. 2022 Nov 7;21:236. doi: 10.1186/s12933-022-01671-w (PMC9641859; doi:10.1186/s12933-022-01671-w)
Supplement: Supplementary file 1 — Additional file 1: Fig. S1. Flow-chart of the studied participants. Fig. S2. Cumulative incidence of cardiovascular and non-cardiovascular mortality in the whole population and according to glucose tolerance. Table S1. Cox-proportional hazard model evaluating the association between NT-ProBNP levels and all-cause mortality according to baseline HbA1c levels by excluding patients with chronic kidney disease. [file 12933_2022_1671_MOESM1_ESM.pdf]

Supplementary Figure S1 Flow-chart of the studied participants.

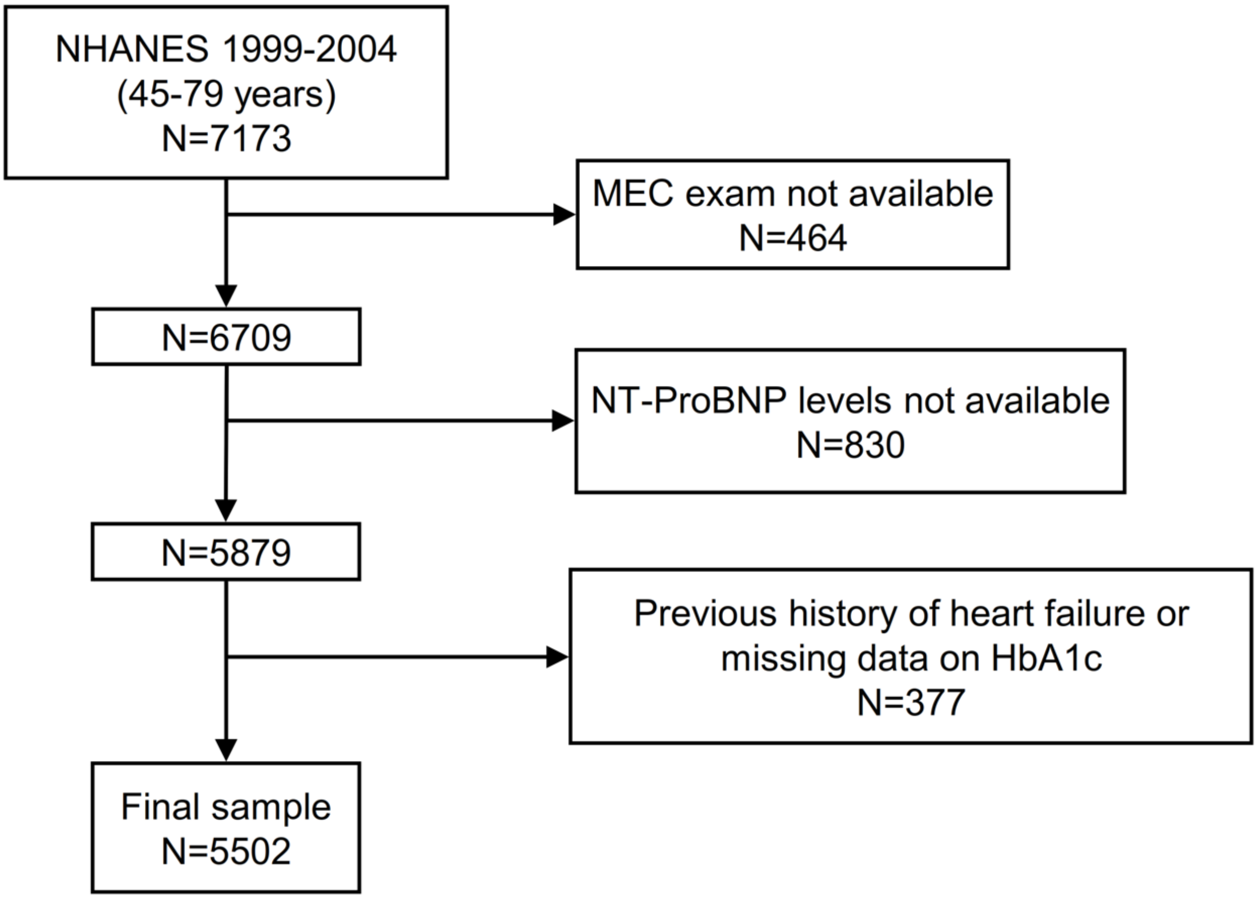

Abbreviations: NHANES, National Health and Nutrition Examination Survey, MEC, mobile examination center.

Supplementary Figure S2 Cumulative incidence of cardiovascular and non-cardiovascular mortality in the whole population and according to glucose tolerance.

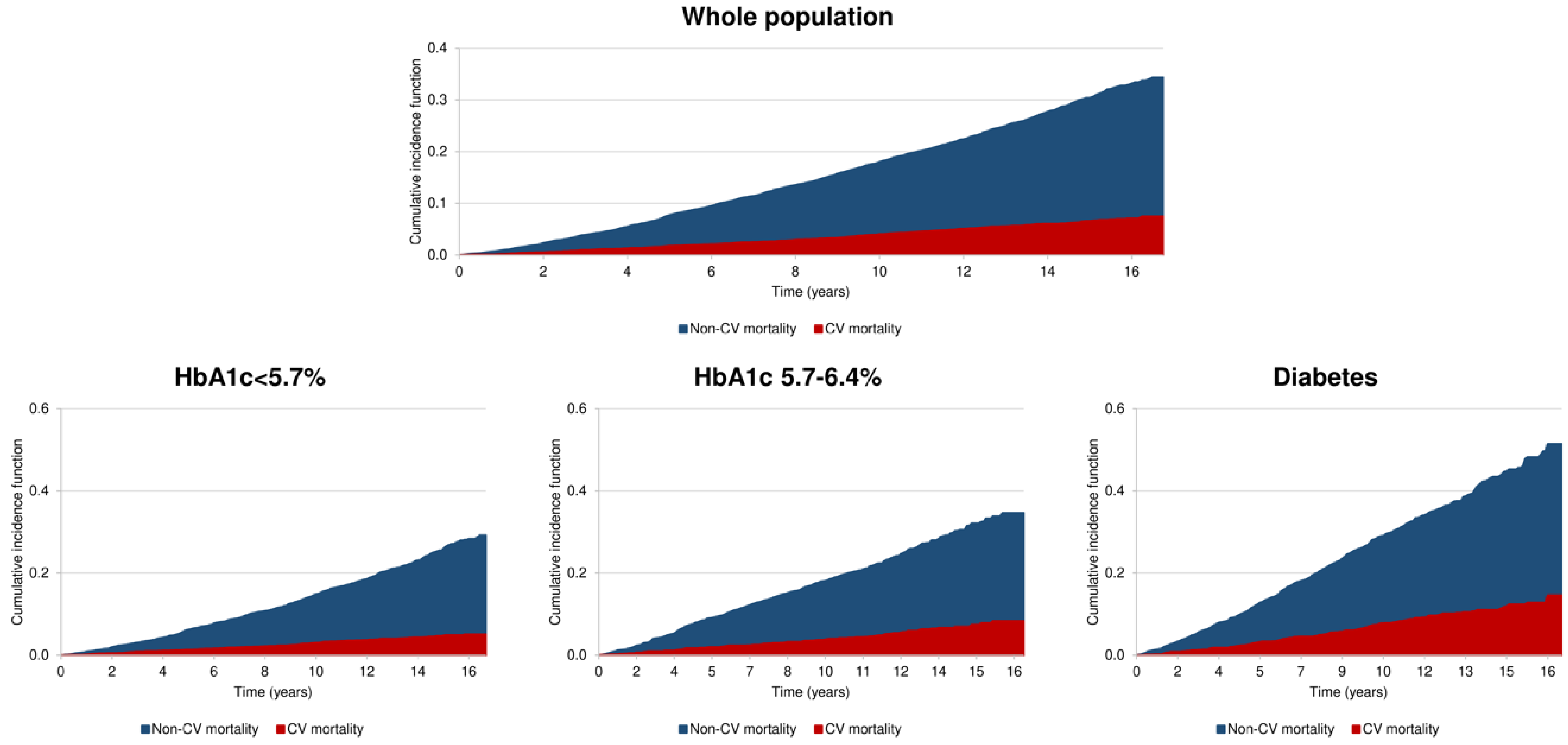

Supplementary Table S1 Cox-proportional hazard model evaluating the association between NT-ProBNP levels and all-cause mortality according to baseline HbA1c levels by excluding patients with chronic kidney disease.

Population	NT-ProBNP (pg/ml)	All-cause mortality		
		HR	95% CI	p-value
Whole population	<100	1.00	Ref.	
	100-299	1.37	1.12-1.67	0.003
	≥300	2.04	1.53-2.73	<0.001
HbA1c <5.7%	<100	1.00	Ref.	
	100-299	1.31	0.98-1.73	0.065
	≥300	1.76	1.22-2.54	0.003
HbA1c 5.7-6.4%	<100	1.00	Ref.	
	100-299	1.39	0.93-2.09	0.103
	≥300	2.51	1.75-3-61	<0.001
Diabetes	<100	1.00	Ref.	
	100-299	1.29	0.97-1.73	0.082
	≥300	2.11	1.44-3.11	<0.001

Covariates included in model were age, sex, race-ethnicity, body mass index, education, cigarette smoke, total cholesterol, eGFR, blood pressure category, prevalent cardiovascular disease and alcohol consumption.

Abbreviations: HR: hazard ratio; CI, confidence interval; NT-ProBNP, N-terminal pro-B natriuretic peptide.
